# The Future of Healthcare Management: A Social Medicine Perspective

**DOI:** 10.3390/ijerph22060861

**Published:** 2025-05-30

**Authors:** Ilaria Tocco Tussardi

**Affiliations:** Department of Diagnostics and Public Health, Section of Hygiene, University of Verona, 37134 Verona, Italy; ilaria.toccotussardi@univr.it

In the evolving landscape of global health, the traditional boundaries between clinical care and social contexts are becoming increasingly porous. As health systems grapple with complex and intersecting crises—ranging from rising chronic disease burdens to health inequities and demographic shifts—the perspective of social medicine offers a compelling lens through which to envision a new paradigm for healthcare management [1]. This article outlines a vision for the future of healthcare that fully integrates social medicine, reorienting systems to prioritize health equity, community engagement, and socially informed decision-making.

## 1. From Pathology to Person: Shifting the Focus of Management

Historically, healthcare management has often been shaped by institutional logic centered on efficiency, resource containment, and clinical outcomes. While these aspects remain undeniably important, they can sometimes overlook the upstream social determinants of health that shape individual and community wellbeing. In this context, social medicine—by placing social, economic, and environmental factors at the forefront—advocates for a shift in how health systems are structured, evaluated, and governed [1].

This broader perspective suggests that healthcare managers need to look beyond the walls of hospitals and clinics. It calls for collaboration with other sectors such as urban planning, education, labor, and civil society organizations. In this model, healthcare management becomes more than just an administrative function; it serves as a bridge between clinical interventions and social transformation.

Integrating social determinants into management is not only a matter of ethical responsibility, but can also be a practical strategy. Evidence from initiatives such as the Gesundes Kinzigtal model in Germany illustrates how community-centered approaches can lead to better health outcomes and cost savings by addressing factors like income, housing, and social isolation [2].

## 2. Envisioning a Socially Integrated Health System

This vision leads us to imagine a health system where management decisions are guided not only by financial metrics or service volume, but also by indicators of social cohesion, housing stability, food security, and mental well-being. In such a system, community health workers are integrated into the organizational framework, and health budgets are co-designed with local communities based on participatory health assessments.

Advances in technology and data science can play a crucial role in this transformation. Digital platforms, for example, can track social vulnerabilities in real time, enabling more equitable resource distribution. Artificial intelligence (AI) could help detect patterns of exclusion in access to care. However, it is important that these tools are developed within a robust ethical and social framework to avoid exacerbating existing disparities [3]. Social medicine can provide the necessary framework to ensure that these technologies serve all communities equitably.

Moreover, electronic health records might evolve into more integrated social health platforms that incorporate data from sectors like housing, employment, and education, thus creating a more comprehensive patient profile. Programs such as the Camden Coalition in the U.S. have already shown that integrating social data into healthcare planning can reduce hospital readmissions and improve the continuity of care [4].

Real-world examples from diverse contexts further illustrate how the integration of social medicine principles into healthcare management can take shape across settings and scales. Initiatives such as social prescribing in the U.K. [5], community-based palliative care in Kerala [6], Medicaid-supported social interventions in North Carolina [7], and intersectoral governance strategies in Barcelona [8] highlight the tangible impact of socially informed approaches to care and policy (see Table 1).

## 3. Governance and Policy: From Participation to Co-Design

When considering governance, a social medicine-informed vision of healthcare management also points to the limitations of traditional top-down policy models, which may struggle to meet the diverse needs of contemporary populations. A shift toward participatory governance seems essential—where communities are not simply consulted, but actively involved in co-designing services [9].

Such a governance model recognizes lived experience as a valuable form of expertise, ensuring that the perspectives of marginalized groups are considered when setting healthcare priorities. This also calls for a broader approach to managerial training, where healthcare managers are equipped not only with knowledge of finance and logistics, but also with an understanding of sociology, ethics, and community engagement.

Furthermore, financing models will need to evolve. The growing interest in health impact bonds, for instance, points to new ways of aligning social investments with health outcomes. These innovative approaches underscore the importance of management structures that are capable of assessing long-term, intersectoral impacts, rather than simply focusing on short-term service delivery metrics [10].

## 4. Future Directions: From Scenario to Transformation

Looking ahead, several key trends could define the future of healthcare management shaped by social medicine:

1. Health as an Intersectoral Responsibility: Health policies will be co-designed alongside education, housing, and labor policies. Local health agencies could evolve into “Health Equity Hubs”;

2. Ethically Guided Technology: AI and predictive tools will be employed within frameworks of community oversight and transparency;

3. Transformative Managerial Training: Future healthcare managers will be trained in participatory methods, human rights, and social change, alongside traditional subjects such as economics and epidemiology;

4. Social Impact Metrics: New performance indicators will evaluate community well-being (e.g., food access, social isolation, and housing stability), in addition to clinical outcomes.

## 5. The Role of Public Health Education in Advancing Change

Public health education institutions play a pivotal role in preparing the next generation of healthcare managers to navigate socially complex health systems. To truly integrate social medicine into healthcare management, academic programs must evolve beyond the traditional curricula. Instead, interdisciplinary training that includes sociology, ethics, political science, and community organizing is essential. Universities and schools of public health could serve as incubators for socially informed leadership, offering experiential learning opportunities that engage students directly with marginalized communities. Moreover, by embedding co-design principles and participatory evaluation methods into coursework, institutions can model the very governance structures they seek to promote. Partnerships between academia, health systems, and civil society can also foster translational research that informs real-world policy and managerial practice. In this way, public health education is not only a site of skill acquisition, but also a driver of cultural and institutional change.

## 6. Conclusions and Policy Implications: From Vision to Action

In conclusion, while the integration of social medicine into healthcare management is still an ongoing process, its potential is already evident in numerous local and international initiatives. From community-based palliative care models in Kerala, India, to the implementation of social prescribing in the U.K., these examples highlight the possibilities that emerge when healthcare systems are aligned with social realities.

The future of healthcare management involves balancing technical efficiency with social accountability, and clinical precision with human solidarity. By embracing the principles of social medicine, we can begin to build health systems that are not only more effective, but also more just.

Policymakers are encouraged to integrate social metrics into national health performance indicators, invest in interdisciplinary training for health managers, and create institutional frameworks for community co-governance. Ultimately, investing in socially integrated healthcare management is not just ethically right, it is economically viable and contributes to systemic resilience.

## Figures and Tables

**Table 1:** Examples of integration of social medicine principles into healthcare management.

Initiative	Location	Description	Key Outcomes/Insights
Social prescribing	United Kingdom	The National Health Service links patients to community service and activities (e.g., walking groups or art classes)	Improved mental well-being: fewer emergency department visits
Community-based palliative care	Kerala, India	A volunteer-driven model supported by professionals provides accessible end-of-life care	Demonstrates community involvement and the de-medicalization of care
Health opportunities pilots	North Carolina, USA	Medicaid reimburses for social services such as housing support, food assistance, and transport	Early evidence of improved social determinants and reduced hospital use
Salut als Barris	Barcelona, Catalonia, Spain	City-wide strategy integrates health equity into various sectors (e.g., planning, housing, and transport - “Health in All Policies” model)	Offers a governance model for embedding social medicine into policy
